# Successful Conservative Management of Aplasia Cutis Congenita in a Preterm Neonate

**DOI:** 10.7759/cureus.87096

**Published:** 2025-07-01

**Authors:** Aleksandre Gvaladze, Tamar Gvalia, Konstantine Kvaratskhelia, David Gagua, Tinatin Gagua

**Affiliations:** 1 Obstetrics and Gynecology, Gagua Clinic, Tbilisi, GEO; 2 Neonatology, Gagua Clinic, Tbilisi, GEO; 3 Obstetrics and Gynecology, David Tvildiani Medical University, Gagua Clinic, Tbilisi, GEO

**Keywords:** acc, aplasia cutis congenita, congenital skin defects, friedens aplasia cutis, neonatal dermatology, rare congenital conditions, type 5 aplasia cutis congenital

## Abstract

Aplasia cutis congenita (ACC), type 5, is considered a rare condition and is typically recognized by symmetric skin defects visible at birth. Due to the limited number of documented cases, there are no established management guidelines, which can result in the use of unnecessary surgical or antibiotic interventions that may expose newborns to avoidable risks. We present a case from Gagua Clinic in Tbilisi, Georgia, involving a newborn with large, symmetric lesions on the trunk and around the umbilicus, which were managed conservatively and showed complete re-epithelialization over the course of a few months. This case highlights the successful conservative management of ACC with extensive skin defects, achieved without the need for surgery or antibiotics.

## Introduction

Aplasia cutis congenita (ACC) is a rare condition, affecting approximately 5.10 per 100,000 births [1]. First described by Cordon in 1767 [2], ACC is typically diagnosed at birth and is characterized by a well-demarcated, transparent membrane that lacks skin and underlying tissue. While the majority of cases (96%) involve the scalp [1], lesions can also occur on other parts of the body, including the face, feet, and abdomen, either as isolated findings or in combination. Due to the small size of some lesions (<0.5 cm) [3] and their heterogeneous distribution, the condition is likely underreported. The etiology is believed to be multifactorial and may involve genetic predisposition [4], chromosomal abnormalities [5], uteroplacental dysfunction, teratogenic factors, or other unknown causes. In 1923, ACC was classified into nine types [6], based on the affected body region, associated abnormalities, and inheritance patterns.

Management strategies range from conservative approaches to surgical interventions such as skin flaps or grafts [7]. However, most cases are managed conservatively and demonstrate a favorable prognosis with spontaneous re-epithelialization. The occurrence of ACC type 5 in premature newborns is exceptionally rare, and available data on optimal management in this vulnerable population remain limited. This case is particularly significant as it demonstrates the feasibility and positive outcome of conservative, non-surgical treatment in a preterm neonate with extensive skin lesions. By documenting this case, we aim to contribute meaningful insight into the management and prognosis of this uncommon and challenging clinical condition.

## Case presentation

The mother, a G1P1 woman in her mid-20s, had no significant genetic or family history. Her medical history included a recent hysteroscopy-polypectomy for abnormal uterine bleeding and a bicornuate uterus. In the same year, she conceived a monochorionic-biamniotic twin pregnancy. At 11-12 weeks of gestation, one twin demised in utero.

A detailed anatomy scan performed at 20 weeks revealed a small-for-gestational-age fetus with an abdominal circumference of 135 mm (4.4th percentile). Umbilical artery (UA) Doppler and amniotic fluid index (AFI) were within normal limits. At 26 weeks, uncomplicated fetal growth restriction was diagnosed with an abdominal circumference of 184 mm (<2.5th percentile), although Doppler and anatomy scans remained normal, and the AFI was measured at 9 cm.

Growth assessments were conducted every two weeks, with weekly monitoring of UA and middle cerebral artery (MCA) Doppler values. At 33+5 weeks of gestation, the mother developed hypertension with a blood pressure of 150/100 mmHg. A 24-hour urine collection revealed proteinuria of 0.5 g/24 h, leading to a diagnosis of preeclampsia and initiation of antihypertensive therapy.

At 34+1 weeks, she experienced multiple episodes of severe hypertension, with blood pressure readings ranging from 160/110 to 180/110 mmHg. This necessitated induction of labor and administration of magnesium sulfate for seizure prophylaxis. At the time of induction, UA and MCA Doppler values were normal, the estimated fetal weight was 1700 g (ninth percentile), the AFI was 10 cm, and the biophysical profile score was 8/10.

The first and second stages of labor were uneventful. A vigorous female neonate was delivered at 34 2/7 weeks, weighing 1500 g, with Apgar scores of 7 and 9 at one and five minutes, respectively. The third stage of labor was actively managed with controlled cord traction. Gross examination of the placenta revealed thrombosed regions and absent or fragmented cotyledons, prompting manual removal of retained placental tissue and membranes.

**Figure 1:**
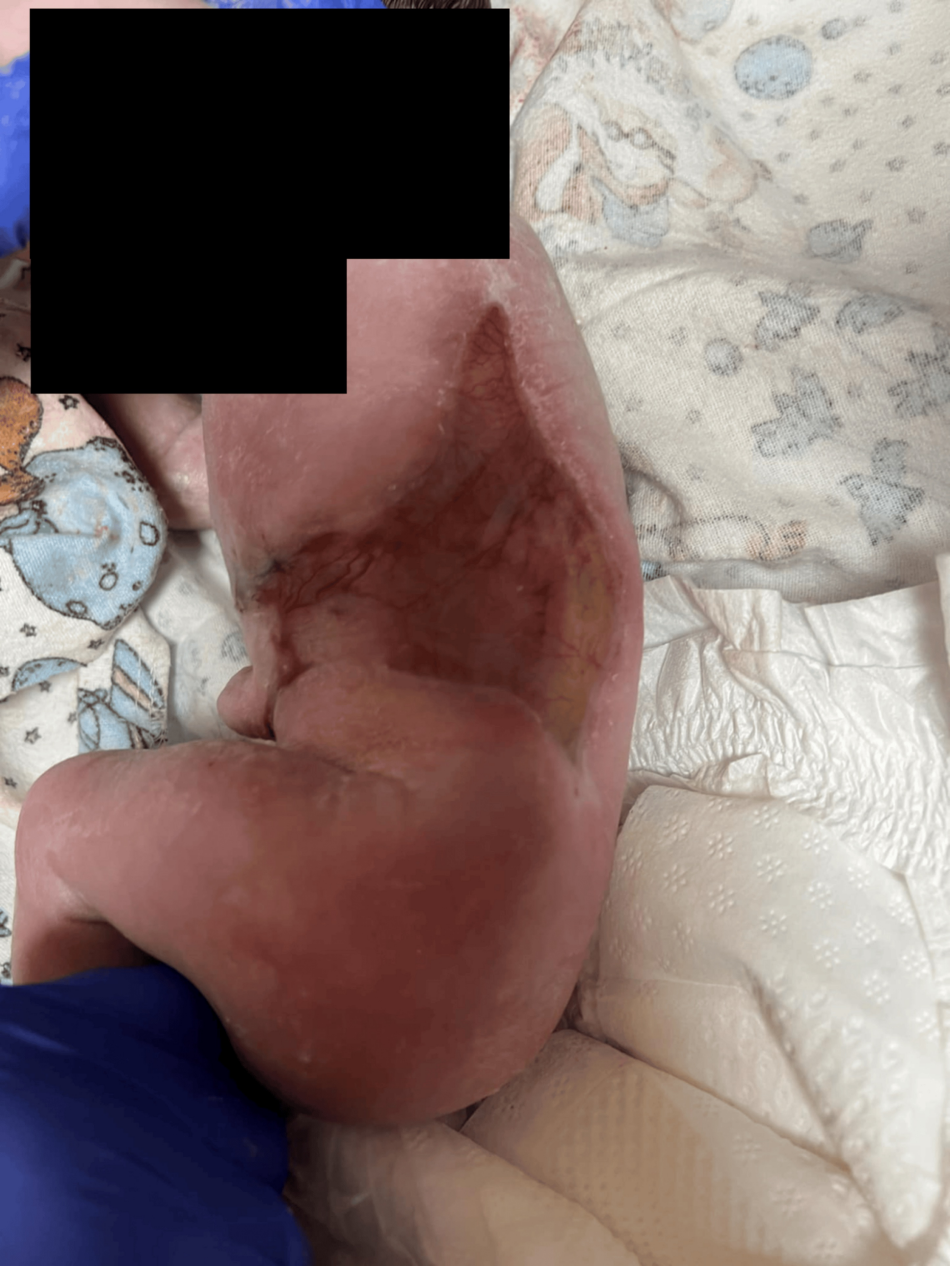
Left side of the trunk immediately after birth showing a skin lesion covered by a translucent membrane

The newborn presented with symmetric, well-demarcated “H”-shaped skin defects on the abdomen and trunk, measuring 12 × 6 cm. The defects were covered by a translucent membrane through which the internal organs and ribs were visible (Figure 1).

The patient was diagnosed with abdominal ACC and classified as type 5 according to Frieden’s classification. Postnatal abdominal and cranial ultrasound examinations revealed no abnormalities, and infection markers were negative. The primary goals of management were to prevent infection, constriction, and evisceration of abdominal contents while promoting effective wound healing.

Wound care involved the use of a stabilized super-oxidized solution containing reactive oxygen species, primarily hypochlorous acid, for cleansing. Following this, a hydrogel composed of water, sodium chloride, and hypochlorous acid was applied directly to the wound using sterile gauze. Dressings were initially changed every three hours for the first four days and then every four hours until the formation of scar tissue. Electrolyte levels remained within normal limits, and no signs of infection were observed throughout the treatment.

**Figure 2:**
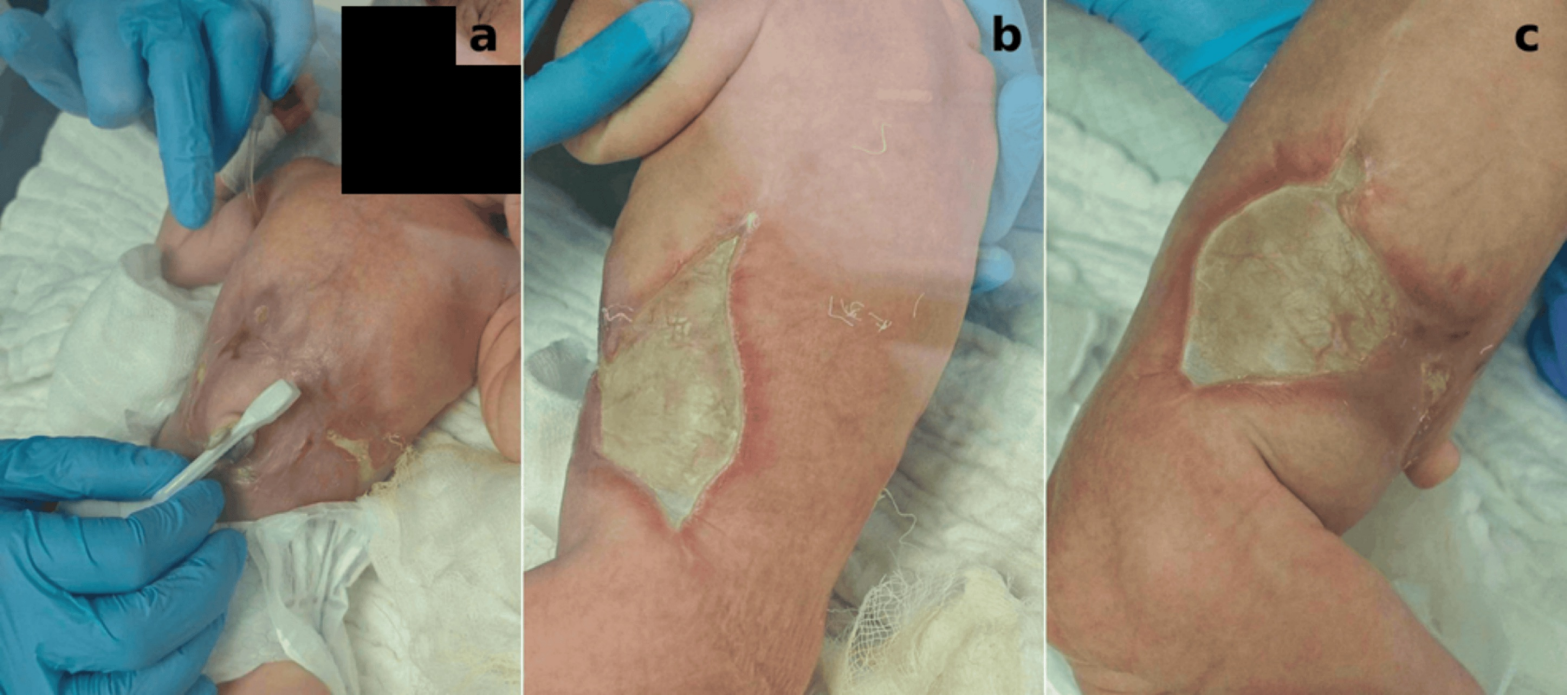
Skin defect on day 10 showing a noticeable reduction in size and early signs of re-epithelialization: (a) abdomen around the umbilicus, (b) left trunk, and (c) right trunk

**Figure 3:**
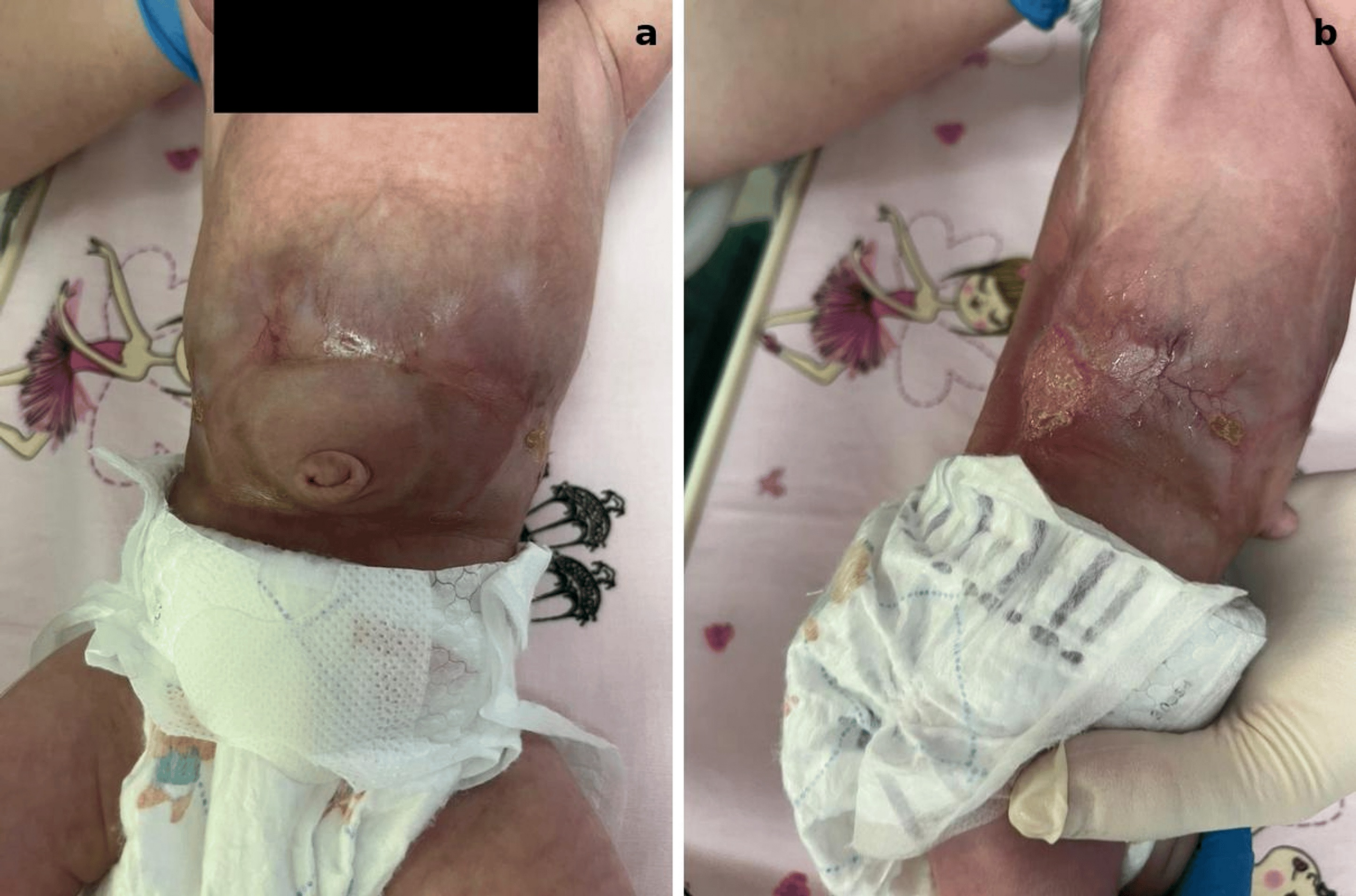
Lesions at four weeks showing significant improvement with substantial scar tissue formation covering more than half of the affected area: (a) abdominal region and (b) right lateral trunk

**Figure 4:**
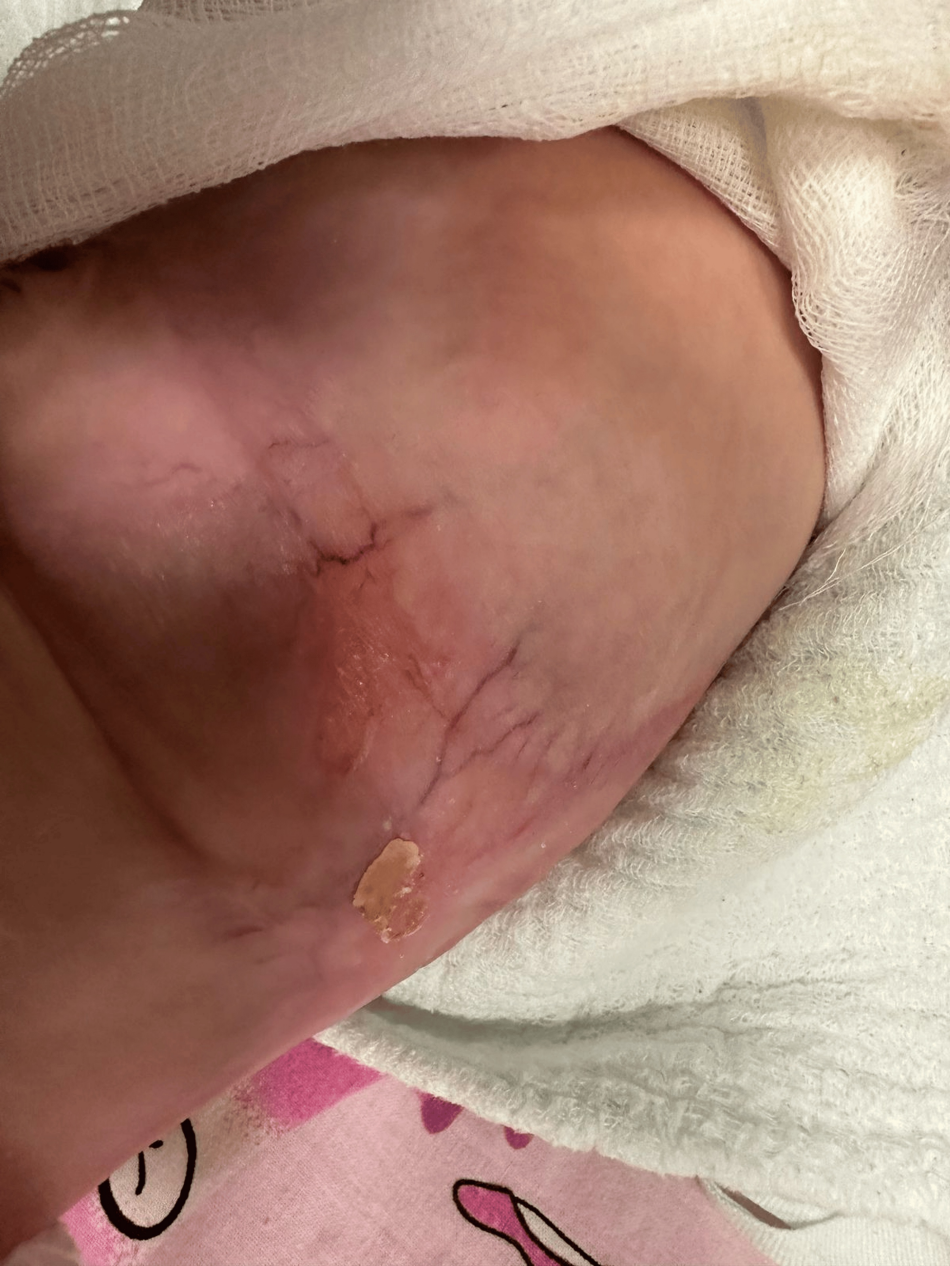
Left side of the lesion at eight weeks showing near-complete re-epithelialization with only minimal residual lesion

During the first 10 days, the skin defects demonstrated progressive epithelialization and noticeable reduction in size, with the development of hairless scar tissue (Figure 2). By the fourth week (Figure 3), the central portion of the lesion had completely healed, while the remaining defects along the flanks continued to improve. At eight weeks (Figure 4), significant healing had occurred, leaving only a small residual lesion and smooth, hairless scar tissue over most of the affected area. By the ninth week, complete re-epithelialization was achieved, and the patient was discharged in stable condition with full recovery.

## Discussion

ACC is a rare congenital condition. Frieden’s widely used classification system categorizes ACC into nine types based on the anatomical location of the lesions and associated anomalies of the neonate and placenta. Type 5 ACC, which is associated with fetus papyraceus or placental infarction, is characterized by multiple symmetric lesions that may appear on the chest, scalp, flanks, axillae, or extremities [6]. Other abnormalities linked to type 5 ACC include a single UA, developmental delay, spastic paralysis, clubbed hands or feet, and amniotic bands. In our case, there was documented fetal demise at 11-12 weeks of gestation, and the surviving neonate presented with symmetric, “H”-shaped lesions on the abdomen and trunk, clinical features consistent with type 5 ACC.

To date, only about 100 cases of type 5 ACC have been reported [8]. The condition shows an even distribution between males and females [9], with a predominance among monochorionic-monoamniotic twins. However, cases have also been described in monochorionic-diamniotic twins, triplets, and even sextuplets [10]. The incidence of type 5 ACC is likely rising due to the increasing use of in vitro fertilization (IVF), which has contributed to a higher rate of multiple gestations [11]. This trend is particularly relevant in Georgia, which is emerging as a growing medical tourism destination for IVF.

The pathogenesis of type 5 ACC remains incompletely understood, though it is thought to be closely related to vascular anastomoses between twins, present in approximately 90% of monochorionic-diamniotic and monochorionic-monoamniotic pregnancies, and to a lesser extent, some dichorionic-diamniotic pregnancies. Two main hypotheses have been proposed. The first is the disseminated intravascular coagulation (DIC) hypothesis, which suggests that the intrauterine death of one twin may initiate a DIC cascade in the surviving twin, leading to thromboembolic events, ischemia, and necrosis in areas of vulnerable fetal vasculature, typically affecting the abdomen, trunk, and extremities. The second is the acute hypotension and transfusion hypothesis, which proposes that a rapid transfusion of blood from the surviving twin to the demised co-twin through placental vascular connections results in acute hypotension and hypoperfusion, ultimately causing necrosis in regions supplied by terminal vessels. Further research is needed to clarify the exact mechanisms underlying this condition.

It is also noteworthy that type 5 ACC is frequently associated with placental abnormalities, oligohydramnios, and low birth weight, findings that suggest a suboptimal intrauterine environment. These factors may contribute to the failure of in utero healing, even though spontaneous resolution of lesions before birth is theoretically possible [12].

There is currently no standardized consensus on the management of ACC. Treatment is generally individualized based on the size, depth, and location of the lesion. Surgical intervention with skin grafting is often considered for larger or complicated lesions [7], though this approach carries risks such as hemorrhage, graft necrosis, and infection. Skin grafts are typically recommended for scalp lesions involving exposed bone, given the elevated risk of hemorrhage and meningitis [13]. Additionally, large lesions or those causing significant fluid or electrolyte imbalances may warrant surgical intervention. Morrow et al. proposed a conservative treatment regimen similar to that used for burn patients, including the use of sulfadiazine and petroleum gauze [14].

In the case presented here, despite the substantial size of the lesions, there were no signs of electrolyte imbalance or infection. A conservative approach was pursued, consisting of regular moisturizing to prevent desiccation and infection while promoting natural healing. Prophylactic topical antibiotics were not used. Over time, this strategy led to complete re-epithelialization without complications.

The overall prognosis of ACC is generally favorable, with most lesions healing spontaneously within a few weeks. Residual scarring may occur, sometimes requiring future plastic surgical intervention. While many small scars are barely noticeable, mortality rates associated with large ACC lesions range from 20% to 50% [15].

## Conclusions

ACC is a rare congenital disorder that remains poorly understood. Large lesions are associated with complications such as electrolyte imbalance, constriction, and infection, and may require surgical intervention to prevent adverse outcomes. We presented a case of a preterm neonate with type 5 ACC who exhibited extensive lesions on the abdomen and trunk. These were successfully managed with conservative treatment, avoiding the risks associated with surgery and systemic antibiotics. This case demonstrates the effectiveness of non-invasive management strategies for type 5 ACC in the neonatal population. However, due to the rarity of this condition, further data are needed to establish standardized treatment protocols. Until such guidelines are developed, management should remain individualized, taking into account the patient’s clinical condition and the specific characteristics of the lesion.
